# Treating postnatal depressive symptoms in primary care: a randomised controlled trial of GP management, with and without adjunctive counselling

**DOI:** 10.1186/1471-244X-11-95

**Published:** 2011-05-27

**Authors:** Jeannette Milgrom, Christopher J Holt, Alan W Gemmill, Jennifer Ericksen, Bronwyn Leigh, Anne Buist, Charlene Schembri

**Affiliations:** 1 Department of Psychology, Psychological Sciences, University of Melbourne, Victoria 3010, Australia; 2 Parent-Infant Research Institute, Department of Clinical & Health Psychology, Heidelberg Repatriation Hospital, Austin Health, 300 Waterdale Road, Heidelberg West, Victoria 3081, Australia; 3 Northpark Hospital, Victoria, Australia; 4 Department of Medicine, University of Melbourne, Victoria, Australia

## Abstract

**Background:**

Postnatal depression (PND) is under-diagnosed and most women do not access effective help. We aimed to evaluate comparative management of (PND) following screening with the Edinburgh Postnatal Depression Scale, using three best-practice care pathways by comparing management by general practitioners (GPs) alone compared to adjunctive counselling, based on cognitive behavioural therapy (CBT), delivered by postnatal nurses or psychologists.

**Methods:**

This was a parallel, three-group randomised controlled trial conducted in a primary care setting (general practices and maternal & child health centres) and a psychology clinic. A total of 3,531 postnatal women were screened for symptoms of depression; 333 scored above cut-off on the screening tool and 169 were referred to the study. Sixty-eight of these women were randomised between the three treatment groups.

**Results:**

Mean scores on the Beck Depression Inventory (BDI-II) at entry were in the moderate-to-severe range. There was significant variation in the post-study frequency of scores exceeding the threshold indicative of mild-to-severe depressive symptoms, such that more women receiving only GP management remained above the cut-off score after treatment (p = .028). However, all three treatment conditions were accompanied by significant reductions in depressive symptoms and mean post-study BDI-II scores were similar between groups. Compliance was high in all three groups. Women rated the treatments as highly effective. Rates of both referral to the study (51%), and subsequent treatment uptake (40%) were low.

**Conclusions:**

Data from this small study suggest that GP management of PND when augmented by a CBT-counselling package may be successful in reducing depressive symptoms in more patients compared to GP management alone. The relatively low rates of referral and treatment uptake, suggest that help-seeking remains an issue for many women with PND, consistent with previous research.

**Trial Registration:**

The study is registered at ClinicalTrials.gov, Trial Registration Number NCT01002027.

## Background

Postnatal depression (PND), defined as an episode of major or minor depression occurring in the first 12 months postpartum, has a point prevalence of 13% at 3 months postpartum [1] and early intervention is indicated to prevent long-term impact on women, their partners and infants [2]. Universal assessment of PND is becoming best practice in many countries around the world [3-5]. Whilst assessment methods recommended vary (e.g., psychometric screening questionnaires, case-finding questions), these developments in practice will see increasing numbers of cases of PND identified, making widespread availability of effective PND care pathways a pressing public health issue in many countries.

General Practitioners (GPs) and postnatal nurses are key primary care professionals engaged with mothers during the postnatal period. It is therefore important to determine whether best-practice management of PND in primary care can offer an effective pathway resulting in alleviation of depression for the majority of women.

Further as many women are reluctant to take antidepressants during lactation, due to potential side effects on the newborn [6] readily available non-pharmacological treatments are essential. Systematic and meta-analytic reviews support the efficacy of psychological therapy for PND [7,8]; however, there have generally been too few studies included to draw conclusions about the relative effects of various types of psychological treatments. Nevertheless, cognitive-behavioural therapy (CBT) is clearly one of the most effective treatments for depression at other life stages [9].

Whilst CBT is generally delivered by mental health specialists such as psychologists, some evidence for the ability of nurses to deliver psychological interventions for PND in primary care has been published. However, studies conducted to date have not explicitly compared such interventions to management by GPs. To our knowledge, in the postnatal period, five controlled trials have evaluated psychologically-informed interventions delivered by primary care practitioners (generally nurses) [10-14]. Only one study [14] has compared non-specialists with specialists (allocation to specialists versus non-specialists was not random).The interventions were CBT-based or counselling-based (psychodynamic therapy was also evaluated in one study), and the nurses were trained in these approaches. With the exception of one study [12], nurse delivered interventions were shown to be more effective in the short-term than routine care (which consisted in most cases of standard nursing practices in place for perinatal women). Morrell et al. [11] also found that benefits for women in the intervention group were maintained at 12 months postpartum. Interestingly, Cooper et al. [10] found an expertise effect, such that women treated by non-specialists showed significantly greater reduction in depressive symptoms compared with those treated by specialists (however treatment allocation was not randomised).

Effective and manualised psychological interventions can be successfully translated to widespread delivery by a range of primary care professionals and could be a valuable resource for health systems around the world. For example, in Australia, the advent of the National Perinatal Depression Initiative (NPDI [15]) will see the implementation of universal screening for perinatal mood disorders. As a large number of depressed women will be identified following screening, it is important to establish which primary care pathways commonly provided in most countries can provide effective treatment of PND. Assessment without evidence-based treatment being readily available raises duty of care issues and, in isolation from other service improvements, screening for depression in primary care will generally be ineffective in reducing morbidity or improving outcomes [16].

The present study similarly sought to examine the effectiveness of counselling informed by the principles of CBT and delivered by primary care practitioners to women with PND. In addition, this study sought to address currently unanswered questions: Is the same treatment delivered by different professionals similarly effective (e.g. trained nurses versus psychologists)? In this RCT we compare three model care pathways: management by trained GPs alone and management by trained GPs augmented with a counselling-CBT intervention delivered either by a trained nurse or a psychologist.

## Methods

### Sample & Procedures

The study (Trial Registration Number NCT01002027) took place in three municipalities in Melbourne, Australia with approval from Austin Health Human Ethics Research Committee. Postnatal women with infants < 12 months of age were screened by nurses working in primary care at maternal child health centres during regular routine visits. The Edinburgh Postnatal Depression Scale (EPDS) [17], is a simple 10-item questionnaire designed to screen for symptoms of PND. The EPDS has good acceptability [18] and is used worldwide. Women scoring ≥13 on the EPDS were invited into the study. Once baseline data were secured, a woman's GP was contacted and offered training, prior to their first patient being allocated to one of the three study groups. Inclusion criteria were: screening score above cut-off on the EPDS; infant aged 6 weeks to 4 months. Exclusion criteria were: insufficient English; psychotic symptoms; need for immediate crisis management. Having been trained in diagnosis and management of postpartum mood disorders (see next section), GPs were asked to conduct a diagnostic assessment on all women to confirm that their patients were depressed and would require treatment. A coded, variable-length permuted blocks allocation schedule was pre-generated by an independent person and administered centrally by administrative staff. Women were randomised with a 1:1:1 allocation ratio to the three groups. At entry, each participant agreed to randomization to either treatment by the GPs themselves, or with adjunctive sessions with a nurse or a psychologist. Irrespective of group allocation all women were asked to schedule at least 3, fortnightly check-up visits with their GP and all participants remained under the overall care of their own GP.

#### Training

Each participant's GP received brief, focussed training, consisting of a face-to-face session with a psychologist in the GP's practice (about 45-60 minutes), supported by detailed printed materials, to enhance their ability to manage PND. This involved systematically working through a 25-page training manual covering screening, diagnosis with standard psychiatric criteria (DSM-IV), risk assessment and management, engagement, a biopsychosocial model of PND, medication during lactation, common patient concerns, onward referral and principles of treatment (including supportive counselling strategies and cognitive-behavioural strategies). Telephone consultation with a psychiatrist was available to provide additional advice on medication for PND. A GP-specific, one-page PND Management Guide (developed by *beyondblue*; available at http://www.beyondblue.org.au/index.aspx?link_id=7.102) was also provided. GPs were free to prescribe antidepressant medication in all three groups (as in other RCTs of psychological interventions for PND in primary care settings [11]). A total of 46 GPs received the training (some had more than one of their patients in the study).

Twenty two nurses completed a half-day training workshop in the counselling-CBT intervention [19]. The training drew on an evaluated CBT program for PND [20,21] adapted for routine application in primary care using a counselling framework. The training was conducted by a senior psychologist, with several years experience in delivering CBT for PND, and covered three phases of the intervention: assessment, goal setting and treatment, addressing the key skills and therapist pitfalls in each stage. The sessions focussed on: psycho-education about PND, goal setting and problem solving, behavioural interventions (e.g. encouraging pleasant activities, relaxation) basic cognitive techniques (e.g. link between thoughts and feelings, challenging unhelpful beliefs and thoughts). Additional components included: the partner relationship, social support and the mother-baby relationship. The Overcoming Postnatal Depression manual [19] provided detailed step-by-step, prompted, six-session content. The psychologists delivered the same intervention package.

#### Treatment Groups

##### Group A: GP management

Women allocated to this group were managed as usual by their own GP (trained in PND management).

##### Group B: Adjunctive counselling-CBT from a nurse

Women allocated to this group received six sessions (one per week over six weeks) of the manualised Overcoming Postnatal Depression Program. This counselling-CBT program was delivered by a trained nurse at maternal and child health centres and was an adjunct to GP management.

##### Group C: Adjunctive counselling-CBT from a psychologist

Women allocated to this group received six sessions (one per week over six weeks) of the same Overcoming Postnatal Depression Program as group B. This counselling-CBT was delivered by an experienced psychologist at a hospital Psychology department. Again this was delivered as an adjunct to GP management.

#### Outcome Measures

The main outcomes were levels of depressive symptoms and the proportion of participants with symptoms below the cut-off score indicative of mild to severe depressive symptoms. Two validated measures of depressive symptoms were used and were administered at baseline, again after 3 weeks, and immediately post-study. The Beck Depression Inventory II (BDI-II [22]) was the main measure. The BDI-II is a well-validated, 21-item self-report questionnaire that provides a clinical measure of depressive symptoms and threshold scores for classifying symptoms into minimal, mild, moderate and severe categories. The BDI-II has good internal consistency (α = 0.91) and good test-retest reliability (r = .96).The short form of the Depression Anxiety and Stress Scales (DASS 21 SF) [23] was used to monitor levels of stress and anxiety, which commonly occur co-morbidly with depression. The stress and anxiety scales have alpha values of 0.81 and 0.73 respectively [23].

In addition, women completed questionnaires rating the perceived effectiveness of treatment on binary (Yes/No) and Likert-type (1 to 10) scales. Information on medication use was collected post-study. As all outcome measures were self-report, it was not possible to obtain blinded measures of symptomatology.

### Power & Sample Size

Based on the average baseline BDI-II score in a previous study (BDI-II = 23.8, SD = 8.4) a post-treatment improvement of 30% (7.1 points) would take average scores to the midpoint of the "mild" range of depressive symptoms (BDI-II = 14-19). Applying these numbers we calculated: n = 2(0.84 + 1.96)^2 ^(8.4/7.1)^2 ^= 22.0, at 80% power with p = 0.05. We therefore continued recruitment until at least n = 22 had been achieved in all 3 groups.

### Statistical Analysis

The BDI-II score classifications given by Beck et al [22] were used to categorise cases as either above (score ≥14 = mild, moderate or severe depressive symptoms) or below threshold (score < 14 = zero or minimal depressive symptoms). Between-group differences were tested by Analysis of Covariance (ANCOVA) controlling for baseline scores. We asked if GP management differed from adjunctive counselling-CBT *per se*, and also whether there was a difference between counselling-CBT by psychologists compared to nurses. This required two, *a priori*, orthogonal contrasts as follows: Contrast i) ***Group A vs [Group B + Group C]/2***. Contrast ii) ***Group B vs Group C***.

The primary analysis was by intention-to-treat [24] using maximum likelihood imputation of missing values (expectation maximisation: EM). All computations were carried out in SPSS 16.

## Results

### Participants at Baseline

**Figure 1:**
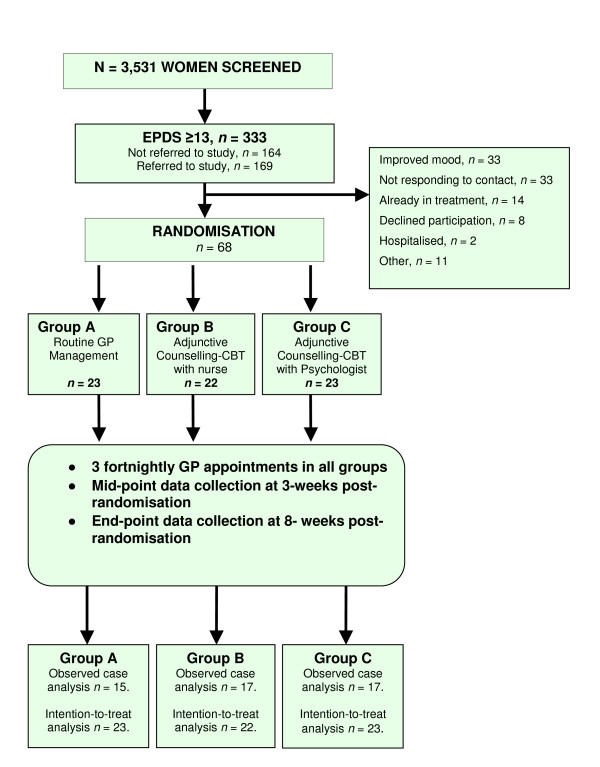
Participant Flowchart.

Figure 1 shows the flow of participants through the study. Of 3,531 women screened, 333 scored ≥ 13 on the EPDS. One hundred and sixty four of these women were not referred to the study. Reasons for non-referral by nurses were not recorded systematically. However, the reasons for non-participation among those referred to the study are detailed in Figure 1. Ultimately, sixty eight women were randomised. The mean baseline EPDS of these 68 women (16.98, SD 4.49) was not significantly different from the 101 referred women not randomised (16.36, SD 3.56). Twenty-three women were allocated to Group A (GP management), 22 to Group B (adjunctive counselling-CBT with nurse) and 23 to Group C (adjunctive counselling-CBT with psychologist). Table 1 shows baseline characteristics of each group. As is appropriate in a RCT, no between-group significance tests were conducted on baseline values [24,25]. Mean baseline scores on the BDI-II were in the moderate to severe range for all groups indicating the presence of clinically significant depressive symptoms. For the 66 women in total the average BDI-II score at baseline was 29.14 (SD 10.12) with scores ranging from 12 to 51 points. Group averages are given in Table 2.

**Table 1:** Baseline Characteristics of Participants

	Treatment Condition A (GP) (*n *= 23)	Treatment Condition B (GP+ nurse) (*n *= 22)	Treatment Condition C (GP+ psychologist) (*n *= 23)
**Mean Screening EPDS (SD)**	17.1 (4.5)	16.8 (4.8)	17.0 (4.5)

**Mean Mothers' Age (SD)**	30 (3.3)	33.1 (4.4)	31.4 (5.6)

**Mean Infant age in weeks (SD)**	17.03 (9.22)	14.84 (11.44)	20.68 (9.15)

*Marital Status, n (%)			
Married/De Facto	18 (85.8%)	20 (90.9%)	21 (91.3%)
No partner	3 (14.3%)	2 (9%)	2 (8.6%)

*Born In Australia, n (%)	17 (81%)	19 (90.5%)	21 (91.3%)

*English Speaking, n (%)	21 (100%)	19 (90.5%)	21 (91.3%)

*Education, n (%)			
High School only	5 (23.8%)	3 (13.6%)	7 (30.4%)
Degree or Higher	15 (71.4%)	14 (63.6%)	14 (60.9%)

*Income, n (%)			
< $40,000	7 (33.4)	4 (18.1%)	2 (8.6%)
$40,000-80,000	11 (52.3%)	1 (45.4%)	12 (52.1%)
> $80,001	2 (9.5%)	5 (22.7%)	6 (26.1%)
Wouldn't divulge	1(4.8%)	3 (13.6%)	3 (13%)

*Number of Children, n (%)			
1	13 (61.9%)	8 (36.4%)	9 (39.1%)
2	5 (23.8%)	10 (45.5%)	11 (47.8%)
> 2	3 (14.3%)	4 (18.2%)	3 (13%)

*indicates missing data on these variables

**Table 2:** Baseline and post-study depressive symptoms

	*Treatment Condition A* *(Routine management)*	*Treatment Condition B* *(CBT-counselling with nurse)*	*Treatment Condition C* *(CBT-counselling with psychologist)*
Mean Baseline BDI-II (SD; 95% CI)	27.9 (10.8; 23.3-32.6)	25.5 (8.3; 21.7-29.3)	30.9 (10.7; 26.2-35.6)
Mean Post-study BDI-II (SD; 95% CI)	11.8 (9.8; 6.4-17.2)	6.1 (4.8; 3.7-8.6)	10.9 (11.0; 5.2- 16.5)
*Mean Adjusted Post-study BDI-II (SD; 95% CI)	11.0 (8.0; 7.6-14.5)	6.7 (4.3; 4.8-8.6)	10.4 (9.5; 6.3-14.5)

*Estimate from intention-to-treat ANCOVA controlling for baseline symptoms with maximum likelihood imputation of missing values.

#### Compliance

Seventy one percent of GP appointments were kept (67%, 87% and 67% in groups A, B and C respectively). Similarly, attendance at the 6 counselling-CBT sessions averaged 4.6 and 4 sessions for groups B and C respectively. Of the 68 participants, 50 returned post-study questionnaires. This attrition was demonstrably random with respect to group (χ^2 ^= 1.59, *df *= 2, *p *= .45).

#### Symptoms of depression, anxiety and stress

**Figure 2:**
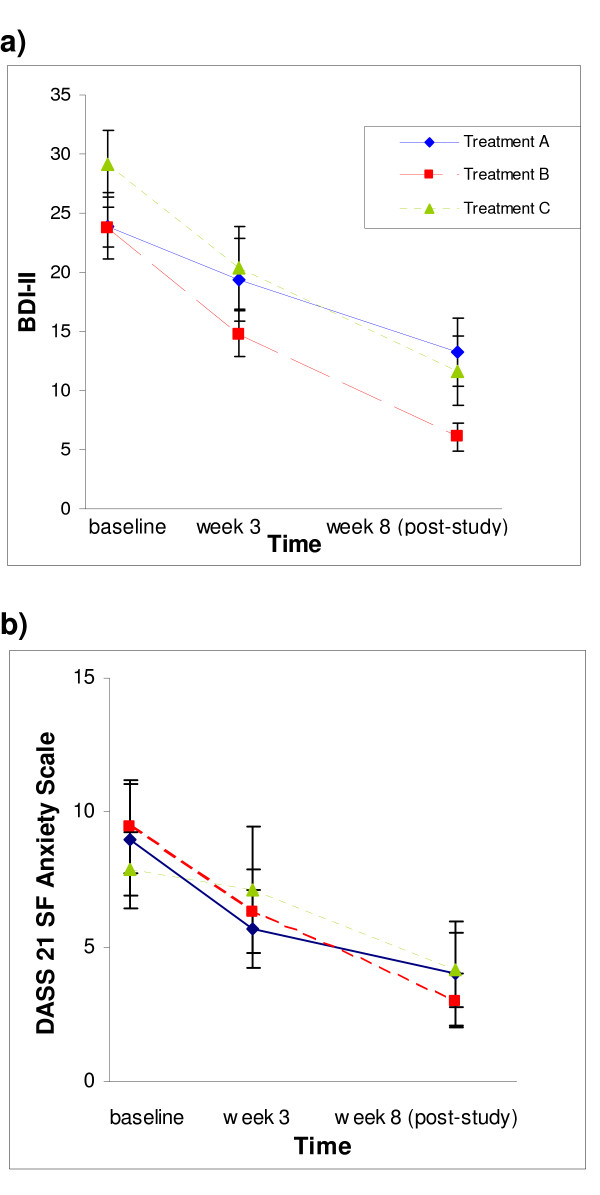
**Changes in Symptoms of Depression and Anxiety**. **a)** Beck Depression Inventory II; **b)** DASS 21 SF Anxiety Sub-scale. For each measure the means of the three groups are plotted across time. Only those cases with complete data are shown:- Group A (GP management), *n *= 12; Group B (Counselling-CBT with nurse), *n *= 12; Group C (Counselling-CBT with psychologist), *n *= 12. Error bars are ± 1 SE.

Graphical inspection of Figure 2a shows that BDI-II scores across all treatments dropped on a similar trajectory. This constituted a significant drop between baseline and post-study (mean reduction in BDI-II scores for all treatment groups combined = 17.3 points, 95% CI 14.2-20.5). Table 2 gives the mean baseline and post-study BDI-II scores for each treatment group. The results of the intention-to-treat contrasts of post-study BDI-II scores controlling for baseline scores showed that variation between treatments was non-significant (*F*= 1.051, *df *= 2,45, *p *= .358). Neither planned Contrast i) *GP management versus counselling-CBT*, nor planned Contrast ii) *Adjunctive counselling-CBT from nurse versus psychologist*, were significant (*p *= 0.347 and *p *= .247 respectively).

Figure 2b shows the significant (p < 0.05) overall drop in anxiety over the course of the study. Similarly to the results for BDI-II scores, there were no significant between-group differences in post-study scores for the three DASS 21 SF scales of *depression*, *anxiety *and *stress *(p > 0.05).

#### Depressive symptoms above threshold

An observed-case frequency analysis of remittance rates based on categorising BDI-II scores as above or below threshold (Table 3), found that the frequency of above-threshold cases did vary significantly post-study, such that those women in GP management (Group A) appeared more likely to exhibit symptoms of depression (Table 3, χ^2^, *df *= 2, *p *= .028). The same information is re-expressed in terms of Relative Risk at the bottom of Table 3. Management in Group B (adjunctive counselling-CBT from a nurse) lowered the risk of an above-threshold outcome relative to GP management, but as numbers are small these findings should be interpreted cautiously.

**Table 3:** Baseline and Post-study frequencies of depressive symptoms

	*Treatment Condition A* *(Routine management)*	*Treatment Condition B* *(CBT-counselling with nurse)*	*Treatment Condition C* *(CBT-counselling psychologist)*
**Baseline**			
*below: above threshold	1:22	2:19	0:21

**Post-study**			
*below: above threshold	8:7	16:1	13:4

**Absolute Risk **(probability of being above threshold post-treatment)	0.47	0.06	0.24

**Relative Risk **compared to Routine management (95% CIs)	1 (reference value)	0.13 (0.02 -0.91)	0.5 (0.18-1.4)

*For frequency analysis, BDI-II scores ≥14 were classed as 'above-threshold'

#### Services accessed and Medication Use

There was a poor return rate from women regarding other services accessed and medication use with only one third of the sample returning these questionnaires. Based on the available data there was no difference in post-study outcome between women known to be taking antidepressants (mean BDI-II score = 10.3, 95% CIs 6.4 - 14.1) and all other women (mean BDI-II score = 9.0, 95% CIs 5.6 - 12.3).

#### Participant Ratings

Forty six women responded to the questions on treatment efficacy. A majority in all groups indicated that treatment was sufficient (9/14, 16/18 and 12/14 in groups A, B and C respectively). On a scale of 1 to 10, respondents rated perceived effectiveness of their treatment highly in all groups (6.9, 8.6 and 7.4 respectively in groups A, B and C), and significantly more highly in group B (Kruksall Wallis test, p = 0.04).

## Discussion

This study compared three pathways of care for managing PND, all treatments requiring training the key primary care health professionals involved. An important question in the management of perinatal mood disorders is whether different "real world" care pathways actually result in amelioration of depressive symptoms, and whether they differ consistently in efficacy [26]. On average, women who were offered GP management in the present study had similar improvements in symptoms of depression and anxiety to those receiving adjunctive counselling-CBT *per se*. Possibly, the GP training component made any additional effect of adjunctive counselling-CBT more difficult to detect. Nonetheless, we also found that women in GP management continued to exhibit a higher frequency of above-threshold depressive symptoms post-study. These data may suggest that adjunctive counselling-CBT involving either psychologists or nurses could be a promising model of collaborative PND management in primary care.

A number of other positive outcomes were found. Firstly, anxiety, (which is often a co-morbid problem with PND) was also effectively reduced by treatment. Secondly, compliance rates were good and women in all groups showed significant reductions in post-study symptoms of depression. Interestingly, there is some suggestion that adjunctive counselling-CBT was most effective when delivered by nurses. This is consistent with some previous findings on the effectiveness of PND treatment programs delivered by both specialist and trained non-specialist practitioners [11,12,27,14]. In the present study, psychologists worked from treatment rooms in a public hospital whilst nurses conducted the first counselling-CBT session at home and subsequent sessions in a health centre. Conceivably, this difference may have contributed to the possible advantage of counselling-CBT delivered by nurses. Baseline BDI-II scores may also have influenced these results, as they were somewhat higher in group C (counselling-CBT with psychologists).

The study has a number of limitations. First, the sample size was relatively small, and attrition reduced this further at follow-up, limiting our ability to generalise from the results. Second, the "control" group itself involved an enhancement of current care, by training GPs. For ethical reasons it was inappropriate to include a wait-listed control group in this study. However the observed improvements in mood (a drop of 17.3 BDI-II points on average) are of a magnitude at least as large as post-treatment effect sizes observed in studies involving psychological interventions versus routine care for PND [8]. Furthermore, in our previous RCT of psychological treatments for PND [21] we found that, following routine care, symptoms of depression and anxiety were essentially unchanged after 12 weeks. Thus, spontaneous improvement seems an insufficient explanation for the large drop in symptomatology following treatment observed in the present study. Third, GP report of depressive symptoms rather than a standardized diagnostic interview was used for inclusion. However, all GPs were trained in diagnosis according to standard criteria and baseline BDI-II scores in all three groups reflected moderate to severe levels of symptomatology. Furthermore, a single psychologist delivered the intervention, again limiting the generalisability of results. The study is also limited in that no diagnostic procedure was carried out post-treatment, so that the numbers of women meeting diagnostic criteria for a depressive disorder following treatment is not known. Referral to the study was relatively low, and of those referred most either could not be contacted (n = 33) or had experienced improved mood (n = 33). Only 8 women still experiencing low mood and not accessing treatment refused involvement with the study. Lastly, no longer-term follow-up was possible so that long-term maintenance of gains cannot be assessed.

Early intervention for PND is essential due to the negative consequences for women and for their close family members in terms of mental health and child socio-emotional development [28,29]. The results presented here add to a growing body of evidence that following a positive screening result for PND many (indeed most) women do not pursue further options for assessment and treatment. Less than 50% of women affected by PND have been reported by others to access treatment [18,30-32]. In this study, only 20% of those screening positive did so and this may have been partly due to nurse's and women's reluctance to participate in a randomised research study.

Even among those who agreed to referral to this study, most did not ultimately take up treatment, although some cited improved mood or had already accessed other treatment options. Low referral rates to, and participation rates in a particular research study such as this may also reflect the reluctance of women to take part in research.

However, given the current evidence, it seems clear that specific research on how to increase women's engagement with treatment would be valuable. Whilst systematic screening for PND offers one possibility for increasing detection (the first step to accessing treatment) data on the ultimate usefulness of screening programs for PND in terms of increased treatment uptake are still relatively scarce. As has been pointed out elsewhere, the introduction of screening in isolation will have little impact [16,33]. In the only published RCT of screening effectiveness [34] a significant reduction in morbidity was found due to the implementation of screening. The key to effectiveness in terms of improving women's outcomes was to systematically follow up all positive screening results with further clinical assessment for depression and access to effective management. Recent meta-analyses of the effectiveness of depression screening (not just for PND) suggest that it can have its biggest impact on morbidity when deployed as part of a well-coordinated health system effort towards identification and treatment. A clear policy of acting on all positive screening results plus a well-resourced treatment component appear to maximise the usefulness of screening for depressive disorders in general [16] and the effectiveness and cost-effectiveness of PND screening in particular [34-36].

## Conclusions

In summary, for the majority of those who received treatment, all three possible models of care appeared effective. It therefore appears that for the management of moderate-to-severe PND, best practice primary care management routes are effective for the majority of women. GP management coupled with adjunctive counselling-CBT yielded promising results. In practice these models of PND management are deliverable by existing primary care professionals. However, rates of both referral to treatment (51%), and subsequent treatment uptake (40%) were low, suggesting help-seeking remains an issue in clinical practice that needs to be addressed by comprehensive research on methods to overcome this obstacle. Training key primary care professionals and strengthening their collaboration is likely to remain centrally important for improving current treatment pathways for PND following screening, under Australia's National Perinatal Depression Initiative, and for similar universal programs in other countries.

## Competing interests

The authors declare that they have no competing interests.

## Authors' contributions

JM, JE, AG and AB conceived the study. JM, JE, and BL contributed to the design of the GP training. CS and BL delivered the training. CH and BL oversaw data collection and monitored the adherence to study protocols. AG and CH designed and executed data analyses. AG (50%) BL (25%) and CH (25%) wrote a first draft of the manuscript. JM, JE, AG, CS, AB, CH and BL all edited subsequent drafts for important intellectual content and all authors agreed on the submitted version.

## Pre-publication history

The pre-publication history for this paper can be accessed here:

http://www.biomedcentral.com/1471-244X/11/95/prepub
